# Role of p-glycoprotein expression in predicting response to neoadjuvant chemotherapy in breast cancer-a prospective clinical study

**DOI:** 10.1186/1477-7819-3-61

**Published:** 2005-09-14

**Authors:** Chintamani, Jai Parakash Singh, Mahesh K Mittal, Sunita Saxena, Anju Bansal, Ashima Bhatia, Pranjal Kulshreshtha

**Affiliations:** 1 Department of Surgery, Vardhman Mahavir Medical College Safdarjang Hospital New Delhi-110023-India; 2 Department of Radiology, Vardhman Mahavir Medical College Safdarjang Hospital New Delhi-110023-India; 3 Tumor Biology Laboratory, Indian Council Of Medical Research, Vardhman Mahavir Medical College Safdarjang Hospital New Delhi-110023-India

## Abstract

**Background:**

Neoadjuvant chemotherapy (NACT) is an integral part of multi-modality approach in the management of locally advanced breast cancer. It is vital to predict response to chemotherapy in order to tailor the regime for a particular patient. The prediction would help in avoiding the toxicity induced by an ineffective chemotherapeutic regime in a non-responder and would also help in the planning of an alternate regime. Development of resistance to chemotherapeutic agents is a major problem and one of the mechanisms considered responsible is the expression of 170-k Da membrane glycoprotein (usually referred to as p-170 or p-glycoprotein), which is encoded by multidrug resistance (MDR1) gene. This glycoprotein acts as an energy dependent pump, which actively extrudes certain families of chemotherapeutic agents from the cells. The expression of p-glycoprotein at initial presentation has been found to be associated with refractoriness to chemotherapy and a poor outcome. Against this background a prospective study was conducted using C219 mouse monoclonal antibody specific for p-glycoprotein to ascertain whether pretreatment detection of p-glycoprotein expression could be utilized as a reliable predictor of response to neoadjuvant chemotherapy in patients with breast cancer.

**Patients and methods:**

Fifty cases of locally advanced breast cancer were subjected to trucut^® ^biopsy and the tissue samples were evaluated immunohistochemically for p-glycoprotein expression and ER, PR status. The response to neoadjuvant chemotherapy was assessed clinically and by using ultrasound after three cycles of FAC regime (cyclophosphamide 600 mg/m^2^, Adriamycin 50 mg/m^2^, 5-fluorourail 600 mg/m^2 ^at an interval of three weeks). The clinical response was correlated with both the pre and post chemotherapy p-glycoprotein expression. Descriptive studies were performed with SPSS version 10. The significance of correlation between tumor response and p-glycoprotein expression was determined with chi square test.

**Results:**

A significant relationship was found between the pretreatment p-glycoprotein expression and clinical response. The positive p-glycoprotein expression was associated with poor clinical response rates. When the clinical response was correlated with p-glycoprotein expression, a statistically significant negative correlation was observed between the clinical response and p- glycoprotein expression (p < 0.05). There was another significant observation in terms of development of post NACT p-glycoprotein positivity. Before initiation of NACT, 26 patients (52%) were p-glycoprotein positive and after three cycles of NACT, the positivity increased to 73.5% patients.

**Conclusion:**

The study concluded that pretreatment p-glycoprotein expression predicts and indicates a poor clinical response to NACT. Patients with positive p-glycoprotein expression before initiation of NACT were found to be poor responders. Thus pretreatment detection of p-glycoprotein expression may be utilized, as a reliable predictor of response to NACT in patients with breast cancer The chemotherapy induced p-glycoprotein positivity observed in the study could possibly explain the phenomenon of acquired chemoresistance and may also serve as an intermediate end point in evaluating drug response particularly if the adjuvant therapy is planned with the same regime.

## Background

Neoadjuvant chemotherapy (NACT) is an integral part of multi-modality approach for the local and systemic management of locally advanced breast cancer (LABC) [1-5]. However chemoresistance is a major problem and one of the proposed mechanisms for its development is the expression of 170-kDa-membrane glycoprotein (usually referred to as P-170 or P-glycoprotein) encoded by multi drug resistance-1 (MDR-1) gene [6-9]. This membrane glycoprotein acts as an energy dependent pump, which actively inhibits accumulation of certain families of chemotherapeutic agents by extruding them from the cells thus leading to a poor response [8,9].

Immunohistochemical detection using monoclonal antibody for p-glycoprotein has been considered to be a more sensitive method than southern, northern or western blot analysis because even very low number of positive cells or lower grade of expression could be readily recognized [10,11].

In some tumors like leukemia's, multiple myelomas, Hodgkin's lymphomas, soft tissue sarcomas the expression of p-glycoprotein at initial presentation has been found to correlate significantly with refractoriness to chemotherapy thus highlighting its importance as a contributor to chemoresistance [12,13].

Pretreatment detection of p-glycoprotein has not been found to be a useful tool for predicting response before the initiation of chemotherapy in breast cancers as its expression has not been commonly observed in the untreated breast cancer cell lines [9,14,15]. There are only scattered reports in the literature of extraordinarily high incidence of p-glycoprotein expression in untreated breast cancer specimen and its role in the assessment of pretreatment p-glycoprotein expression for predicting treatment failure [15-19].

Against this background a prospective study was conducted using C-219 mouse monoclonal antibody specific for p-glycoprotein to ascertain whether pretreatment detection of p-glycoprotein in patients of LABC could be utilized as a reliable predictor of response to neoadjuvant chemotherapy.

## Patients and methods

After approval by the Institution Review Board and the ethical committee of the hospital, 50 fine needle aspiration cytology (FNAC) proven cases of LABC according to AJCC (American Joint Committee on Cancer) classification were included in the study. The tumor size and the axillary lymph node status were measured clinically and by using ultrasonography. Core biopsy was performed for immunohistochemical estimations of p-glycoprotein and ER, PR status in the biopsy specimen before initiating the chemotherapy. Routine and metastatic work up (total blood count, platelet count), chest radiograph, electrocardiography (ECG) (echocardiography when ECG had a positive finding), liver function tests, bone scan, ultrasonography (USG) of the abdomen, renal function tests were routinely done in all the cases.

Three cycles of FAC regime (cyclophosphamide 600 mg/m^2^, adriamycin 50 mg/m^2^, 5-fluorourail 600 mg/m^2^) were given at three weekly intervals and the patients were assessed both clinically and by USG for response in the form of reduction in breast tumor size and axillary lymph node status. Patey's modified radical mastectomy was performed three weeks after the last cycle and the mastectomy specimen was examined for pathological response, resected margins, axillary lymph nodes, ER, PR status and p-glycoprotein expression (post NACT).

The pathological tumor response was evaluated by size measurement at the time of tumor resection macroscopically and by detecting tumor cell existence (or not) microscopically.

**Clinical responders **were defined as patients with a complete (CR) or partial response (PR) [CR: complete resolution of tumor, PR>50% regression in maximum diameter of initial tumor] after 3 cycles of NACT. Non-responders were patients with a minimal response (MR<50% regression in maximum diameter of initial tumor), no change (NC) or local progression. Pathological complete response (pCR) was defined as absence of any gross or microscopic evidence of residual tumor in the mastectomy specimen i.e. absence of residual invasive or in situ disease following NACT. Its assessment was done irrespective of the clinical response status. Clinical response was taken in to consideration for statistical analysis as the pCR was observed in only seven patients (n = 50).

### Immunohistochemical methods

Biopsy specimen was preserved in buffered formalin solution and five-micron sections were prepared on poly-l-lysine coated glass slides. Sections were deparaffinized in xylene and hydrated in alcohol for 15 minutes. Further, incubation was done in 0.3% hydrogen peroxide in methanol solution for 45 minutes; the slides were washed with citrate buffer and kept in koplin jar with citrate buffer (pH-6) at 200 power in microwave (5–6 pulses). Sections were washed with Tris Buffer Saline (TBS) solution and incubated with blocking antibody (C-219 mouse monoclonal antibody) at 37°C overnight. Dilutions used were 1:20. Sections were washed with TBS solution, incubation done with avidin biotin complex (ABC) at 37°C for one hour and 3,3 Diaminobezidine tetra hydrochloride solution was applied for 3–5 minutes. Counter staining with hematoxylin solution was done for 3–5 minutes. The sections were washed with distilled water, air-dried and mounted using DPX mount.

For p-glycoprotein positive, controls were taken as positive breast cancerous cells and negative controls were taken as test slides without primary antibody. The pattern of positive staining was cytoplasmic. The monoclonal antibody used was C219 antibody (DAKO M3521) [It recognizes an epitope lying in cytoplasmic domain, 200 amino acids long, of the terminal regions of the p-glycoprotein polypeptide].

**Figure 1:**
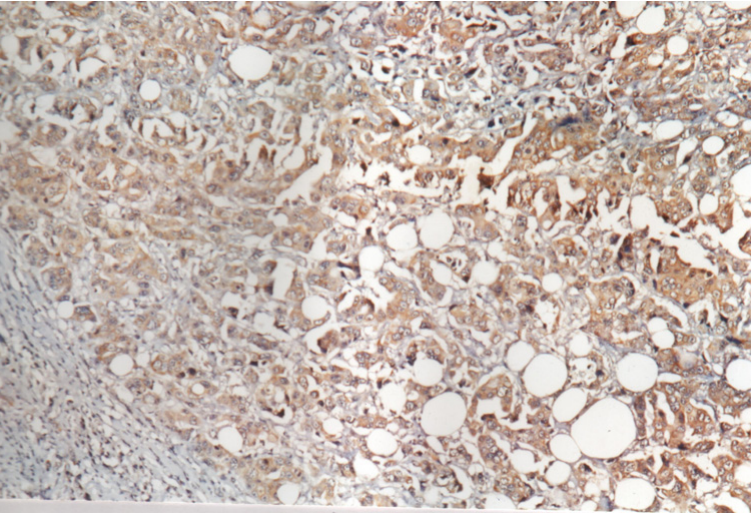
Positive immunoreactivity for p-glycoprotein in breast carcinoma (200×).

**Figure 2:**
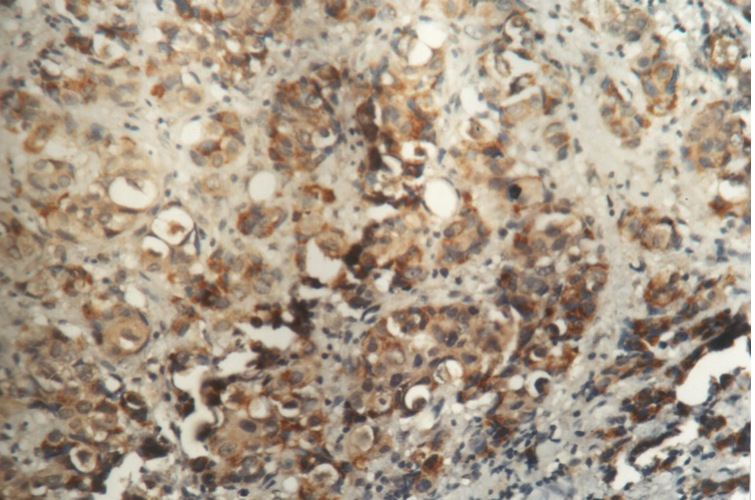
Positive immunoreactivity for p-glycoprotein in breast carcinoma (400×).

**Figure 3:**
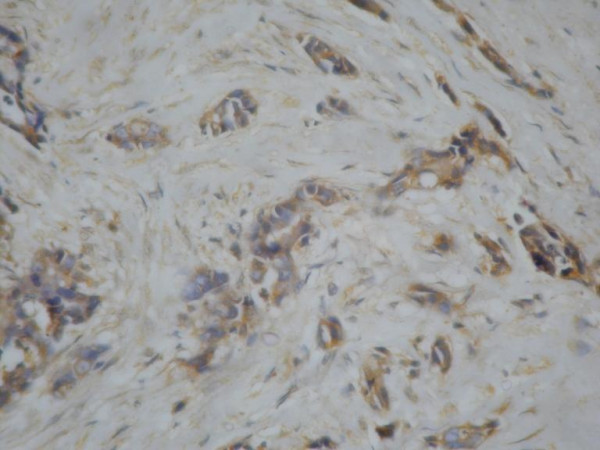
p glycoprotein expression 1+.

**Figure 4:**
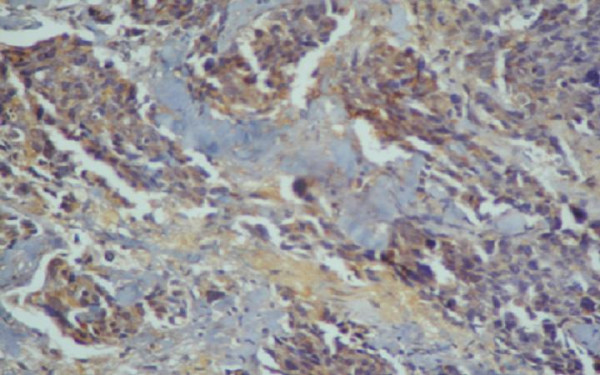
p glycoprotein expression 2+.

**Figure 5:**
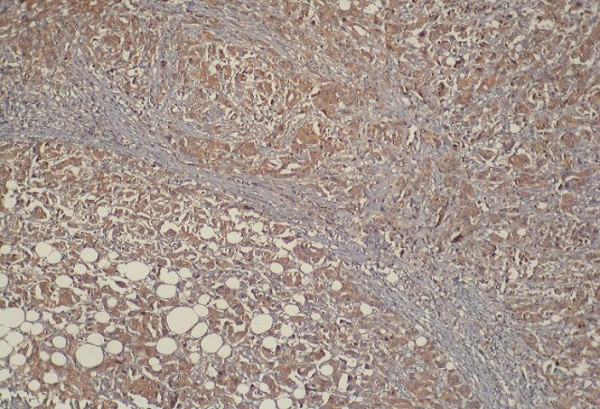
p glycoprotein expression 3+.

**Figure 6:**
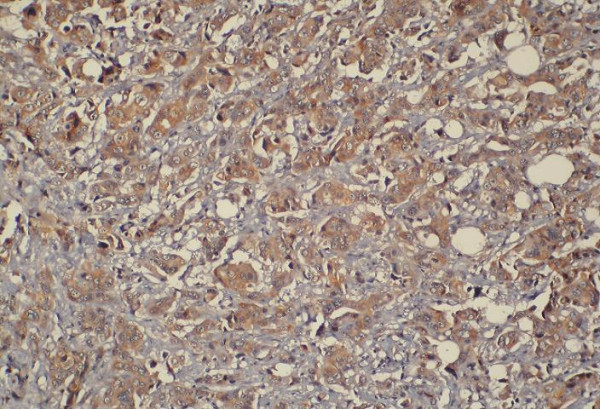
p glycoprotein expression 3+1.

**Figure 7:**
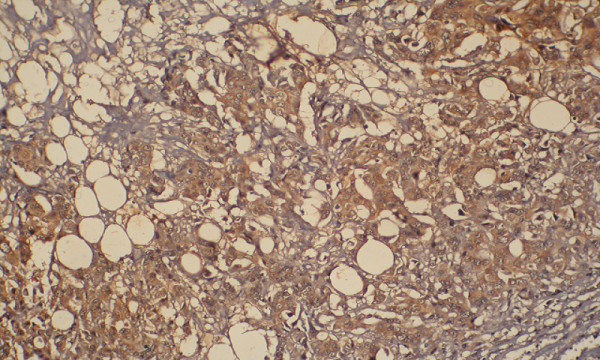
p glycoprotein expression 3+2.

The p-glycoprotein expression was interpreted on the basis of percentage of p-glycoprotein positive cells against total population of cells [**1+**<25%, **2+ **= 25–50 % and **3+ **>50% positive cells] (figure 1, figure 2, figure 3, figure 4, figure 5, figure 6, figure 7).

The intensity of staining was also assessed and it was found that staining intensity correlated closely with percentage of positive cells and a single index i.e. percentage of positive cells was used for analysis.

### Statistics

Descriptive studies were performed with SPSS version 10. The significance of correlation between tumor response and p-glycoprotein expression was determined with chi square test.

## Results

Fifty cases of LABC were included in the study with the mean age being 43 years (range 25–60 years) and 26 patients (53.3 %) were premenopausal. The mean tumor size before NACT was 8 cm. Twenty-four (48%) had N1 disease while 26 patients (52%) presented with N2 disease in the axilla (table 1).

**Table 1:** Tumor size and axillary lymph node status before NACT (n = 50)

**Tumor size (cm)**	**Frequency**	**Percent**
<5 cm	8	16.7
5–8 cm	18_[A1]_	36.7
8–10 cm	13_[A2]_	26.7
10 cm	10	20
**Lymph node status**	(N = 50)	100
N1	24	48
N2	26	52

In the biopsy specimen before initiation of NACT, 26 patients (52%) were p-glycoprotein positive and 24 were negative (n = 50). The distribution of p-glycoprotein positive patients based on grades was 1+ and 2+ in 8 patients each (16%), 3+ in 6(12%) and 4+ in 4(8%) (Additional file 1). Among the premenopausal patients 16 (59.2%) stained positive for p-glycoprotein while only 10 (43.4%) of postmenopausal patients stained positive. The difference was statistically not significant.

The clinical response was assessed using stringent World Health Organization (WHO) criteria and reduction in mean tumor size after three cycles of NACT was found to be statistically significant (p < 0.05)(table 2). Thirty patients (n = 50) were responders [*complete response in 7 and partial response in 23 patients (n = 30)*] while the rest 20 (40%) were non-responders (table 2).

**Table 2:** Mean tumor size before and after NACT (Paired sample statistics)

Status	Mean	(n)	Std.Deviation	Std. Error mean				
Pre NACT tumor size(cm)	8	50	2.7	.50				
Post NACT tumor size(cm)	4.3	50	2.1	.38				
	**Paired differences**					**t**	**df**	**Significance (2-tailed)**
	
Paired samples test	Mean	Std. deviation	Std. error mean	95% confidence interval of the difference				

Pair **1**: Pre NACT tumor size-Post NACT tumor size	3.677	1.6152	.2949	lower 3.074	upper 4.280	12.468	29	0.000

Significant clinical response was observed in the axillary lymph node status after NACT. There was complete response in N1 patients (n = 24) i.e. they were all down staged to N0. Amongst the patients with N2 disease (n = 26), 18(69.2%) were downstaged to N1, the rest 8(31.3%) patients showed no response (table 3). This downstaging in the axillary lymph node status was found to be statistically significant (p < 0.05).

**Table 3:** Axillary lymph node status before and after NACT(n = 50)

N = 50	N0	N1	N2
Before NACT	nil	24(48%)	26(52%)
After NACT	24(48%)	18(36%)	8(16%)

The clinical response however did not show have any significant correlation with the pre NACT tumor size, age and menopausal status of the patients. Only seven patients (14%) showed pCR (pathological response) and significantly all these were p-glycoprotein negative. There was no statistically significant correlation observed between ER status and clinical response in the present study.

When pre NACT p-glycoprotein expression was correlated with the clinical response, it was observed that out of 30 clinical responders 21 patients (70%) were p-Glycoprotein -ve and 9(30%) were p-glycoprotein +ve while out of 20 clinical non-responders 17 patients were p-glycoprotein positive (85%). This was found to be statistically significant and as it was observed that most of the non-responders were p-glycoprotein positive.

Significantly all the seven complete responders were p-glycoprotein negative and out of nine p-glycoprotein positive patients that were partial responders, six showed very low levels of p-glycoprotein expression (1+). Thus, there was a statistically significant correlation observed between the degree of positivity of p-glycoprotein expression and poor clinical response (p < 0.05).

When p-glycoprotein positive patients were analyzed for the grades of positivity and clinical response, it was observed that increase in grade was associated with decreased response rates. There was thus an inverse relation ship observed between p-glycoprotein expression and the clinical response to NACT.

With an increase in the level of p-glycoprotein expression, the response rates dropped significantly. When the clinical response was correlated with pre-NACT p-glycoprotein expression, (With the confidence limit of 99%-p = 0.01, chi square test was applied) a statistically significant negative correlation was observed between p-glycoprotein expression and clinical response

The change in the p-glycoprotein expression before and after NACT was also found to be statistically significant in the present study. Before initiation of NACT 26 patients (52%)were p-glycoprotein positive and after three cycles of NACT, it increased to 73.5%. This chemotherapy induced p-glycoprotein positivity could possibly explain the phenomenon of acquired chemoresistance after NACT and may also serve as an intermediate end point in evaluating drug response particularly if the adjuvant therapy is planned with the same regime.

## Discussion

Carcinoma of breast is a leading cause of cancer mortality in women all over the world and the second most common malignancy in India after carcinoma of the uterine cervix [1,4]. In India like in other developing countries 25–30% cases are locally advanced at the time of diagnosis [1,4]. The recommended approach for the management of LABC is a multimodality approach intended to provide both local and systemic control and studies have confirmed that surgery alone is an inadequate treatment [3]. The realization that patients with LABC are likely to have undetectable micro metastases at diagnosis has lead to systemic treatment assuming an important role, as even aggressive surgical techniques do not reduce the higher incidence of local recurrence. Most importantly surgery does not change the pattern of distant failure in these patients as they often have micrometastatic disease at the time of diagnosis [18-21].

Neoadjuvant chemotherapy was first introduced with a 70% objective response rate in 1970s and was initially utilized to convert unresectable tumors to smaller tumors making them more amenable to local control with either surgery or radiotherapy. Although the correlation between the tumor response and prognosis is still uncertain, it is generally believed that such a relationship may exist [2,16,17]. The other important advantage of NACT is that it provides an *in vivo *chemosensitivity test for assessment of tumor response from which prognostic information could be obtained.

Development of resistance to chemotherapeutic agents is a major and evolving problem and the search for an ideal predictor of response is still on [2]. One of the proposed contributory mechanisms is the expression of p-glycoprotein, which is encoded by a family of three genes (MDR1, MDR2 and MDR3) in rodents and two genes MDR1 (also known as PGY 1) and MDR 3 (also known on PGY3) in humans. The MDR 1 and MDR 2 gene products (class 1 and class II isoforms) are involved in drug resistance, whereas the biological properties of MDR3 gene product with class II, III isoform are not multidrug resistance [17-22]. The p-glycoprotein is a 17 Kda membrane glycoprotein which functions as an energy dependent drug efflux pump leading to poor response due to decreased accumulation of drugs inside the cells.

The electrophoretic methods such as Northern and Western blotting in detecting p-glycoprotein expression with tiny tissue samples containing very small number of p-glycoprotein expressing tumor cells have not proved satisfactory. Therefore immunohistochemical detection of p-glycoprotein using monoclonal antibodies is now widely accepted for detecting even a single p-glycoprotein expressing cell or low levels of expression, which could be missed by electrophoretic analysis [11].

The p-glycoprotein has been reported to be expressed in several normal human tissues also, notably epithelial cells with excretory/secretary functions (kidney, liver, colon), in endothelial cells at several blood tissue barrier sites (brain, testis), in secretary and gestational endometrium, in placental trophoblasts, and in adrenal glands (predominantly in the cortex). p-glycoprotein is also expressed in natural killer cells, lymphocytes, granulocytes, monocytes and in a minority of CD 34+ hematopoetic stem cells. The pattern of distribution of p-glycoprotein in normal humans suggests that its physiological role is to protect cells against xenobiotics and endogenous toxins [23-25].

Tumors arising in organs that normally express high levels of p-glycoprotein, such as kidneys, adrenals or colon are known to be intrinsically resistant to chemotherapy [9]. p-glycoprotein over expression has also been observed in leukemia's, cervix cancer as well as in soft tissue sarcomas [24]. The role of p-glycoprotein in human breast cancer is however unclear. Most of the published data suggests that p-glycoprotein expression in primary breast tumor is not a common phenomenon [23-25].

Sugawara *et al *detected one positive sample out of nine tumor samples using MRK-16 monoclonal antibody (Mab) in a classical immunoperoxidase staining study. In this study only a few tumor cells were stained within the positive specimen [14]. Using C219 MAb in an avidin biotin immunoperoxidase system, Schneider *et al*, tested 23 breast cancer specimens. None, or only minimal reactivity was found in specimens coming from untreated patients (12 cases) [9]. Dixon *et al*, reported no clear positivity for p-glycoprotein out of 26 primary breast tumors using 219 Mab [26] and Hyun C *et al*, used JSB-1 MAb and detected only 6 p-glycoprotein positive tumors out of 23(26%) primary breast tumors [26]. Verrelle *et al*,. used C494 MAb and detected 17 p-glycoprotein positive tumors out of 20 primary breast tumors. There was a significant negative correlation observed between p-glycoprotein expression and clinical response [18]. They had however used fresh frozen tissue unlike in the present study where paraffin embedded tissue was used. Schneider *et al*, [9] used C 494 and reached exactly opposite results to those observed in the study by Verrelle *et al*, [16-18] where the same antibody was used, this could also be because of the fact that they used fresh frozen tissue [9,18]. In the present study c219 CoAb antibody was used and the results obtained were opposite to those of Schneider et al. probably because of the same reasons.

The p-glycoprotein expression in primary breast cancer is therefore not a commonly observed phenomenon and only two reports of extraordinarily high incidence of p-glycoprotein involved in untreated breast cancer specimen, have appeared in the recent past [17,18]. Ro *et al*, had used 219 Mab and reported that intrinsic drug resistance (pretreatment p-glycoprotein positivity) may play a role in the failure of induction chemotherapy in locally advanced breast carcinoma.

In the present study, 26 out of 50 patients were p-glycoprotein positive (52%) and 30 patients (60%) showed clinical response to NACT however out of 30 clinical responders, 21 patients (70%) were p-glycoprotein negative. It was observed that of the 9 p-glycoprotein positive patients that were responders, 6 patients showed very low levels of p-glycoprotein expression (1+). With an increase in p-glycoprotein expression, the response rate was therefore found to drop significantly. The p-glycoprotein positivity correlated inversely with clinical response to NACT in the present study.

There are some published reports of a correlation between p-glycoprotein expression and menopausal status. The premenopausal patients have been found to have a higher p-glycoprotein expression than the postmenopausal patients [26-32]. In the present study however, there was no statistically significant correlation observed between the menopausal status and the p-glycoprotein expression. This discrepancy could have been due to a smaller sample size.

The presence of estrogen receptors provides a molecular basis for the distinction between human breast carcinoma that are responsive to hormone therapy and those that are not [31]. In various studies correlation between p-glycoprotein expression and estrogen receptor status has been found to be unclear [32]. In some studies it was found that patients whose tumors lacked ER had a higher response rate to chemotherapy [26-32]. In the present study no significant correlation between the ER status and the p-glyoprotein expression could be found although the sample size was small and most of the patients were loco regionally advanced.

The reported response rates following NACT vary between 49 to 94 % in various studies and this has been due to use of different chemotherapy combinations given at variable intervals and doses. The reported pCR rate in most studies has been 4–34(%)[2]. The largest trial (National Surgical Adjuvant Breast and Bowel Project NSABP trial B-18) reported a pCR rate of 13 % [2,33-35]. More recently pCR rate of 26–34% have been reported with the use of texanes and the 5-year overall and disease free survival rates were found to be significantly higher in the group whose primary tumor had a pCR than in the remaining responders [2,33-35].

The response rate in our study was 60% while the pCR rate was 14%(n = 7). The overall clinical response rate observed was lower than that reported in some published series (table 4). This could have been due to a smaller sample size or the fact that majority of our patients were locoregionally advanced or because of both these factors.

**Table 4:** Response to NACT observed in various studies (22,27–31)

	Year	Institute	Neoadjuvant chemotherapy used	Number of patients	Response %
De Lena *et al*.[29]	1979	Southeastern cancer study group	FAC	14	80
Morrow *et al*.[23]	1980	Guy's hospital	AV	12	83
Aisner *et al*.[27]	1982	University of Maryland	FAC	27	74
Sataloff *et al*.[30]	1994	Thomas Jefferson University Hospital	CMF	189	85
Swain *et al*.[28]	1995	M.D. Anderson cancer center	FAC	174	88
Singh *et al*.[22]	1996	PGI, Chandigarh India	CMF	38	75.7
Present Study	2003–2004	VMMC, Safdarjang Hospital New Delhi	FAC	50	60

AV: [Adriamycin, Vincristine]

CMF: [Cyclophosphamide. Methotrexate, 5-fluouracil]

FAC [5-fluouracil, Adriamycin, Cyclophosphamide]

There was a significant clinical response observed in the axillary lymph node status after NACT in the present study (table 3). It has also been observed in a recent NSABP B-18 trial where a significant down staging of the axillary lymph node status following NACTwas observed [35-37].

In the study of Kuerer *et al*, conducted at M D Anderson Cancer Center, on 372 cases of LABC the difference in the axillary lymph node status before and after NACT was not found to be statistically significant but a significant correlation has been reported between disease free and overall survival and the pathological response in the axillary lymph node status in various studies [2,38].

There are only few studies reporting an increase in the frequency of p-glycoprotein expression during or after NACT [15,16]. In the present study it was observed that before initiation of chemotherapy 26 patients (52%) were p-glycoprotein positive and after three cycles of NACT, 73.5% patients showed positivity. This increase in frequency of expression after three cycles of NACT could explain the phenomenon of acquired resistance to chemotherapy. The immunohistochemical detection of p-glycoprotein in surgical specimen after NACT would be an available tool to predict the acquired chemo resistance in breast carcinoma. It could also be used as an intermediate end point in determining drug sensitivity for adjuvant treatment, especially when adjuvant chemotherapy is planned with the same regimen as NACT.

## Conclusion

This study highlights the importance of p-glycoprotein expression in predicting response to NACT in breast cancer patients. Patients with positive p-glycoprotein expression before initiation of NACT were found to be poor clinical responders. This pretreatment detection of p-glycoprotein expression may thus be utilized as a predictor of response to NACT. The increased expression of p-glycoprotein induced by the NACT probably explains the phenomenon of acquired resistance to chemotherapy and the detection of post NACT p-glycoprotein can be used as an intermediate end point in determining drug sensitivity for adjuvant treatment, especially when adjuvant chemotherapy is planned with the same regimen as induction chemotherapy. The toxic and ineffective chemotherapy may thus be avoided in non-responders.

## Competing interests

The author(s) declare that they have no competing interests.

## Authors' contributions

**C **was the surgeon in charge who designed the study and standardized the treatment.

**JP, PK **were the postgraduates in charge of the cases and contributed in the study design's

**AB **were responsible for the immunohistochemical analysis and molecular biology of the tissue,

**AsB**: contributed in the dose regulation and statistical analysis of the results.

**MM **was responsible for the ultrasound assessment of the breast to assess the response.

## Supplementary Material

Additional File 1 The tumor response and staining pattern of tumor samples Click here for file
