# Supplementary material for: Role of p-glycoprotein expression in predicting response to neoadjuvant chemotherapy in breast cancer-a prospective clinical study
Source: World J Surg Oncol. 2005 Sep 14;3:61. doi: 10.1186/1477-7819-3-61 (PMC1224882; doi:10.1186/1477-7819-3-61)
Supplement: Additional File 1 — The tumor response and staining pattern of tumor samples [file 1477-7819-3-61-S1.doc]

Additional file 1:

The patient characteristics and staining pattern of tumor samples

| Case No. | Pretreatment P-glycoprotein Evaluation | Tumor response after three cycles |
| --- | --- | --- |
| 1 | 1+ | PR |
| 2 | -ve | MR |
| 3. | 3+ | MR |
| 4. | 4+ | MR |
| 5. | 3+ | MR |
| 6. | -ve | CR |
| 7. | 1+ | PR |
| 8. | -ve | CR |
| 9. | 2+ | PR |
| 10. | 2+ | MR |
| 11. | 3+ | MR |
| 12. | -ve | CR |
| 13. | +1 | PR |
| 14. | 2+ | MR |
| 15. | -ve | PR |
| 16. | -ve | PR |
| 17. | -ve | CR |
| 18. | 3+ | PR |
| 19. | 1+ | MR |
| 20. | 2+ | MR |
| 21. | 4+ | MR |
| 22. | 2+ | MR |
| 23. | -ve | PR |
| 24 | -ve | PR |
| 25. | -ve | PR |
| 26. | -ve | MR |
| 27. | -ve | MR |
| 28. | -ve | PR |
| 29. | -ve | PR |
| 30 | 1+ | MR  Contd… |
| 31 | 4+ | MR |
| 32 | 1+ | PR |
| 33 | -ve | CR |
| 34 | 1+ | PR |
| 35 | 4+ | MR |
| 36 | -ve | CR |
| 37 | 1+ | PR |
| 38 | 2+ | PR |
| 39 | -ve | PR |
| 40 | -ve | CR |
| 41 | 3+ | MR |
| 42 | -ve | PR |
| 43 | -ve | PR |
| 44 | 2+ | MR |
| 45 | -ve | PR |
| 46 | -ve | PR |
| 47 | 2+ | MR |
| 48 | 3+ | MR |
| 49 | -ve | PR |
| 50 | -ve | PR |

Responders[CR: Complete response,PR: Parial response but>50%]

MR: Minimal or no response
